# A thalamic perspective of (un)consciousness in pharmacological and pathological states in humans

**DOI:** 10.1093/braincomms/fcag021

**Published:** 2026-02-28

**Authors:** Dorottya Szocs, Dian Lyu, Andrea I Luppi, Peter Coppola, Rebecca E Woodrow, Guy B Williams, Judith Allanson, John D Pickard, Adrian M Owen, Lorina Naci, David K Menon, Emmanuel A Stamatakis

**Affiliations:** Department of Clinical Neurosciences, University of Cambridge, Addenbrooke’s Hospital, Cambridge CB2 0SP, UK; Division of Anaesthesia, University of Cambridge, Addenbrooke’s Hospital, Cambridge CB2 0SP, UK; Departments of Neurology and Neurological Sciences, Stanford University School of Medicine, Stanford, CA 94304, USA; Department of Clinical Neurosciences, University of Cambridge, Addenbrooke’s Hospital, Cambridge CB2 0SP, UK; Division of Anaesthesia, University of Cambridge, Addenbrooke’s Hospital, Cambridge CB2 0SP, UK; Montreal Neurological Institute, McGill University, Montreal, QC H3A 2B4, Canada; Department of Clinical Neurosciences, University of Cambridge, Addenbrooke’s Hospital, Cambridge CB2 0SP, UK; Division of Anaesthesia, University of Cambridge, Addenbrooke’s Hospital, Cambridge CB2 0SP, UK; Department of Clinical Neurosciences, University of Cambridge, Addenbrooke’s Hospital, Cambridge CB2 0SP, UK; Division of Anaesthesia, University of Cambridge, Addenbrooke’s Hospital, Cambridge CB2 0SP, UK; Department of Clinical Neurosciences, University of Cambridge, Addenbrooke’s Hospital, Cambridge CB2 0SP, UK; Wolfson Brain Imaging Centre, University of Cambridge, Cambridge CB2 0QQ, UK; Department of Clinical Neurosciences, University of Cambridge, Addenbrooke’s Hospital, Cambridge CB2 0SP, UK; Department of Clinical Neurosciences, University of Cambridge, Addenbrooke’s Hospital, Cambridge CB2 0SP, UK; The Brain and Mind Institute, Department of Psychology, The University of Western Ontario, London, ON N6A 5B7, Canada; Institute for Neuroscience, Trinity College Dublin, Dublin D02, Ireland; Department of Clinical Neurosciences, University of Cambridge, Addenbrooke’s Hospital, Cambridge CB2 0SP, UK; Division of Anaesthesia, University of Cambridge, Addenbrooke’s Hospital, Cambridge CB2 0SP, UK; Department of Clinical Neurosciences, University of Cambridge, Addenbrooke’s Hospital, Cambridge CB2 0SP, UK; Division of Anaesthesia, University of Cambridge, Addenbrooke’s Hospital, Cambridge CB2 0SP, UK

**Keywords:** disorders of consciousness, thalamus, unresponsive wakefulness syndrome, minimally conscious state, fMRI

## Abstract

Currently, there is substantial ongoing discussion around the functional role of the thalamus in consciousness. What is missing in the literature, however, is a systematic investigation of the relevance of specific thalamic nuclei in pharmacologically and pathologically altered states of consciousness in humans. Using resting-state functional magnetic resonance imaging in both healthy anaesthetized volunteers (*n* = 16) and patients with disorders of consciousness (*n* = 22), we sought to identify which specific thalamic subregions in both cohorts may be differentially significant for loss of consciousness. Our findings revealed that, among all nuclei, the pulvinar was found to have the strongest functional connectivity change with loss of consciousness following anaesthesia, while demonstrating distinct functional connectivity patterns related to higher-order default mode and executive control networks. Remarkably, in loss of consciousness in disorders of consciousness patients, the ventral-latero-ventral was found to have the strongest connectivity change in comparison with healthy controls, while exhibiting discrete functional connectivity patterns related to higher-order default mode, executive control and frontoparietal networks. Furthermore, we provide evidence that this neural connectivity biomarker in patients also mirrored the changes observed at the behavioural level, which could have clinical implications for targeted deep brain stimulation in therapy for disorders of consciousness.

## Teaser

There is ongoing debate about thalamic contributions to consciousness. Using anaesthesia to experimentally model disorders of consciousness, Szocs *et al*., 2025 demonstrate differential roles for thalamic nuclei and functional changes mirrored at the behavioural level, with therapeutic implications for targeted deep brain stimulation.

## Graphical Abstract

**Graphical Abstract:**
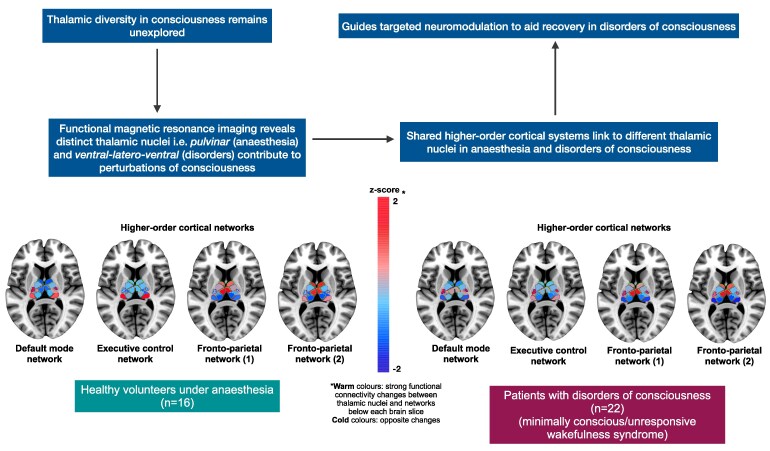

## Introduction

The pursuit of identifying ‘neural correlates of consciousness’ has both scientific and clinical relevance, given the pressing challenge of detecting covert consciousness in uncommunicative individuals with disorders of consciousness (DOC).^1^ We currently still lack a definitive understanding of which neural circuits—common or differential—are responsible for altered states of consciousness and how these work.^2^ There is substantial evidence that functional neuroimaging can not only detect covert awareness in patients who appear entirely unresponsive at the bed side but can predict whether a patient may recover.^3^

Consciousness has been investigated extensively from the perspective of large-scale cortical networks; notably, these major recurrent networks in both health and disease include the default mode (DMN), salience (SN), sensorimotor (SMN), frontoparietal (FPN), dorsal attention (DAN), executive control (ECN), auditory (AN) and visual (VN) networks.^4–11^ Among these networks, the medial prefrontal cortex (mPFC) and PCC (posterior cingulate cortex)/precuneus nodes of the DMN have been found to be significant for predicting conscious awakening.^4,12,13^ Furthermore, salience network (SN) connectivity was able to distinguish between consciouses and unconscious states, whereas the DMN connectivity strength, particularly between the PCC and left lateral parietal cortex (LLPC), instead predicted recovery of consciousness in DOC patients with unresponsive wakefulness syndrome (UWS).^14^ Moreover, regions in the auditory (AN) network were more functionally connected in minimally conscious state (MCS) patients compared to UWS.^15^

There may not be one but, rather, several pathways to unconsciousness, with perhaps some more prominent than others. The thalamus is well-positioned to mediate whole-brain system-level interactions between the cortex, basal ganglia and cerebellum, through inputs of unique thalamic populations—parvalbumin-staining core and calbindin-staining matrix cells—to sustain conscious states,^16,17^ of which the nuclei of the ventral thalamus (specifically the ventral lateral, ventral anterior and mediodorsal nuclei) play a particularly important role in shaping the dynamics of frontal cortical areas.^16^ Moreover, a breakdown of anterior–posterior connectivity has been attributed to the unconscious state induced by anaesthetics, specifically, a depression of the lateral frontoparietal network (FPN).^18^ Precisely how this phenomenon may occur is still unknown, though the authors address possible corticocortical and thalamocortical involvement. On this note, unconsciousness induced by dexmedetomidine has been linked to reduced regional cerebral bloodflow (rCBF) in the default mode network (DMN), fronto-parietal network (FPN) and thalamus,^19^ whereas propofol is known to act upon GABAergic circuits in the thalamus, brainstem and cortex, resulting in the phenomenon of anteriorization or emergence of frontal alpha oscillations.^20,21^ In disorders of consciousness, there have been several studies which attempt to outline thalamic mechanisms in DOC cohorts following brain injury, which focus on the whole thalamus^22^ or the ‘central thalamus’, notably central-lateral (CL) nucleus.^23,24^ At a more subcortical level, coma-causing injuries to the pontine tegmentum in the brainstem have been associated to a functional disconnection of the ventral anterior insula (AI) and pregenual anterior cingulate cortex (pACC),^25^ alluding to a specialized brain network specific to pathological loss of consciousness. Finally, other lines of evidence have suggested multiple pathways to unconsciousness, which highlight the importance of cerebellar and subcortical areas,^26^ ventral tegmentum area^27^ and posteromedial areas.^28^

Emerging evidence points to thalamic involvement in modulating arousal states.^20,23^ This complex subcortical structure has received particular attention as it is situated, both anatomically and functionally, at the convergence of the hypothalamus, telencephalon and brainstem, and most accounts of its functional role propose that it orchestrates dynamic interactions among the cortex, basal ganglia and cerebellum, which together underlie conscious experience.^20,29^ Even more so, at a macroscopic level, it plays a crucial role in systems-level dynamics by integrating multimodal information across large-scale cortical networks.^30^

On a microscopic level, a common classification based on histology divides the thalamus into distinct nuclei: anterior division [i.e. anteriomedial (AM), anteriolateral (AL), anterodorsal (AD), laterodorsal (LD) nuclei]; medial group with midline group [i.e. paratenial (PT), paraventricular (PV), centromedian (CM) nuclei], intralaminar group [i.e. centrolateral (CL), centromedial (CM), parafascicular (PFN) nuclei) and mediodorsal (MD) nuclei]; lateral division [i.e. ventral anterior (VA), ventrolateral (VL), ventral posteriomedial (VPM), ventral posterolateral (VPL) and ventromedial (VM) nuclei]; posterior group [i.e. posteromedial (PM), lateroposterior (LP), pulvinar (PU), medial (MGN) and lateral geniculate (LGN) nuclei]; and a coating of inhibitory neurons (thalamic reticular nucleus).^29^ In an alternative classification scheme, nuclei have been grouped according to the type of input (sensory, limbic or motor),^31^ though these divisions only broadly inform function.^32^ Furthermore, a more nuanced classification model based on thalamocortical output recognizes that individual thalamic nuclei are comprised of an amalgam of cell types,^17,29,33^ most notably the *parvalbumin-staining core* cells, which serve to drive excitatory activity via projections to cortical layers III and IV (receiving glutamatergic inputs from sensory nuclei, association cortical areas and the deep cerebellar nuclei) versus *calbindin-staining matrix* cells, which preferentially project to supra-granular and infragranular cortices in a much more diffuse manner (receive GABAergic inputs from the globus pallidus.^17,20,29,33,34^ Further classification schemes are based on the origin and strength of inputs, i.e. distinguishing first-order from higher-order nuclei.^34^

Importantly, specific thalamic nuclei mediate higher-order cognitive functions,^35^ such as attention^36,37^ and awareness.^38,39^ Emerging animal evidence has demonstrated the involvement of distinct thalamic nuclei in pharmacologically induced unconsciousness, specifically demonstrating that central thalamic stimulation induced arousal in anaesthetized macaques.^38,40^ Equally, human studies have highlighted thalamic interactions with the DMN in anaesthesia and DOC.^28,41^ However, only few human studies have differentiated the cytoarchitecturally and functionally distinct thalamic nuclei, to elucidate their respective relevance in altered states of consciousness.^42–44^ What is missing, therefore, is a thorough investigation of the differential role of individual thalamic nuclei with relevance to pathological impairment in consciousness following brain injury. This is particularly critical in light of the current debate around the functional role of the thalamus in conscious processing.^20,45^ Furthermore, such understanding is crucial from a clinical standpoint, since deep brain stimulation (DBS) of the CL nuclei has been found to improve behavioural outcomes in patients with msTBI^46^ and DOC.^24^ Thalamic stimulation has been implemented in other brain disorders, notably obsessive-compulsive disorder,^47^ Parkinson’s disease, essential tremor and dystonia.^48^ More recently, a stimulation paradigm of both the anterior and medial pulvinar thalamic nuclei has been efficacious for treating drug-resistant epilepsy.^49,50^ Even more so, with advances in MRI spatial resolution and improvements in thalamic parcellation atlases,^51–57^ we are witnessing innovative attempts to functionally disentangle the thalamic ensemble. This has led to more investigations of the role that individual thalamic nuclei play in the context of arousal, most notably in sleep^43^ and propofol anaesthesia,^42^ though the complexity of the thalamus has, so far, been mostly overlooked in the literature in patient cohorts, particularly in disorders of consciousness.

This study provides a systematic investigation of distinct thalamic nuclei, i.e. seven major clusters—pulvinar (Pu), anterior (Ant), medio-dorsal (MD), ventral-latero-dorsal (VLD), central-lateral, lateral-posterior, medial-pulvinar (CL-LP-MPu), ventral-anterior (VA) and ventral-latero-ventral (VLV)—and their functional relationship to cortical and subcortical areas, using functional neuroimaging datasets from cohorts of (i) healthy anaesthetized volunteers (*n* = 16), i.e. pharmacologically induced and (ii) patients with disorders of consciousness (*n* = 22), i.e. pathological, given that anaesthesia has been previously demonstrated to be an experimental model for DOC studies.^26,27,58^ Crucially, we sought to pinpoint which specific nuclei within the thalamus are most strongly associated with loss of consciousness, in both cohorts, which in turn may shed light upon the neural mechanisms of conscious processing, and more crucially, indicate potential areas for targeted brain stimulation therapy in DOC.

## Materials and methods

### Data acquisition

#### Anaesthesia London Ontario dataset

The original study^59^ acquired the anaesthesia dataset between May and November 2014 at the Robarts Research Institute in London, Ontario Canada, have been approved by the Western University Ethics board, and have been previously published.^26,28,60,61^ Nineteen healthy, right-handed, English-speaking volunteers (13 males; age range: 18–40 years) with no reported neurological conditions provided written informed consent. Three participants were excluded due to equipment failure or anaesthetic complications, resulting in a final sample of 16 volunteers.^59^

Resting-state fMRI data were obtained across three propofol-induced conditions: awake (no sedation), deep sedation (Ramsay = 5) and recovery following anaesthesia. Prior to data acquisition in each condition, two anaesthesiologists and one anaesthesia nurse independently verified the Ramsay score (the assessor could not be blinded to the condition as they were responsible for determining anaesthetic depth).^28,59^

Intravenous propofol was delivered via a computer-controlled Baxter AS50 infusion system (Singapore). Dosage was increased in a stepwise manner under computer control until participants reached a Ramsay sedation level of 5, corresponding to an absence of responsiveness to verbal or visual stimulation. When necessary, additional manual adjustments were performed to achieve and maintain the desired propofol concentration, which was stabilized using the pharmacokinetic simulation software *TIVA Trainer* (eurosiva.eu). Predicted blood concentrations followed the Marsh three-compartment model. The initial target concentration was 0.6 µg/ml, with incremental increases of 0.3 µg/ml between assessments of the Ramsay level. This procedure continued until participants ceased to respond verbally and could be aroused only by physical stimulation, marking the onset of data acquisition. Oxygen titration maintained SpO2 above 96%. The mean estimated effect-site propofol concentration was 2.48 (range: 1.82–3.14) µg ml^−1^; the mean estimated plasma propofol concentration was 2.68 (range: 1.92–3.44) µg ml^−1^ and the mean total mass of propofol administered was 486.58 (range: 373.30–599.86) mg.^28,59^ Resting-state fMRI data were collected over an 8-minute acquisition period using a 3-tesla (3T) Siemens Trio scanner. Functional data comprised 256 Echo-planar imaging (EPI) volumes with the following parameters: 33 slices; isotropic resolution = 3 mm; 25% inter-slice gap; TR = 2000ms; TE = 30 ms; flip-angle = 75°; matrix = 64 × 64. Slices were acquired in an interleaved bottom-up order. High-resolution anatomical T1-weighted images were obtained using a 3D MPRAGE sequence (32-channel coil, 1 mm isotropic voxels) with the following parameters: TA = 5 min; TE = 4.25 ms; flip angle = 9°, matrix = 240 × 256.^28,59^

#### Disorders of consciousness dataset

MRI data from 24 patients with DOC were obtained between January 2010 and July 2015 at the Wolfson Brain Imaging Centre Addenbrookes (Cambridge, UK). For this study, the patients were selected from a larger cohort (*n* = 71) on the basis of relatively preserved neuroanatomy integrity.^26,27,58,60^ Patients were admitted to research ward and scanned at Wolfson Brain Imaging Centre. Written informed assent was obtained from the referring clinical teams and a family member or other relevant close contact. Each patient was referred to a full neurological examination and daily behavioural observations. Coma Recovery Scale–Revised (CRS-R) evaluations were conducted at least once on the day of scanning, with additional assessments performed periodically throughout the patient’s hospital stay. For each scanning session, the CRS-R score corresponded to the highest value recorded by the attending physician. Based on these assessments, patients were classified as being in either an UWS or MCS.

Individuals lacking any behavioural evidence of awareness throughout the assessment period were diagnosed with UWS. In contrast, those demonstrating clear behavioural markers of consciousness, such as simple automatic movements (scratching, repositioning bed sheets), sustained visual fixation or pursuit or localization to noxious stimulation, were classified as being in a MCS.^26,27,58^ MCS patients were further classified depending on the presence or absence of language function, into MCS+ or MCS−, respectively.^62^ Detailed demographics are found in Table 1. Ethical approval for data collection was obtained from the National Research Ethics Service.^26,27,58^

**Table 1:** Demographics for DOC patients

Patient	Gender	Age	Aetiology	Months Post-Injury	Diagnosis	CRS	Tennis Task
1	M	21	TBI	45	MCS+	11	positive
2	M	57	TBI	14	MCS−	12	negative
3	M	47	TBI	4	MCS+	10	negative
4	M	36	TBI	34	UWS	8	negative
5	M	17	Anoxic	46	UWS	11	negative
6	F	38	Anoxic	9	MCS−	10	negative
7	F	38	TBI	13	MCS+	11	positive
8	M	29	TBI	68	MCS+	10	positive
9	M	23	TBI	4	MCS+	7	positive
10	F	70	Cerebral bleed	11	MCS+	9	negative
11	F	30	Anoxic	6	MCS−	9	positive
12	F	34	Anoxic	6	UWS	8	negative
13	M	22	Anoxic	5	UWS	7	negative
14	M	37	Anoxic	14	UWS	7	negative
15	F	62	Anoxic	7	UWS	7	negative
16	M	46	Anoxic	10	UWS	5	negative
17	M	21	TBI	7	MCS+	11	negative
18	M	67	TBI	14	MCS−	11	positive
19	F	55	Hypoxic	Unknown	UWS	12	negative
20	M	28	TBI	Unknown	MCS+	8	positive
21	M	22	TBI	Unknown	MCS+	10	negative
22	F	28	ADEM	Unknown	UWS	6	negative

Patients were further subdivided according to their capacity to engage in volitional mental imagery during fMRI scanning, based on a paradigm previously established for detecting covert awareness in patients with disorders of consciousness.^58,63–65^ Two imagery tasks were employed: a motor imagery (‘tennis task’) condition, where patients were instructed to imagine themselves playing tennis, repeatedly swinging their arm to return a ball to a perceived opponent, and a spatial imagery (‘navigation task’) condition, where patients were asked to visualize moving through a familiar environment i.e. around rooms of their house or along well-known city streets, while visualizing the corresponding surroundings. Each paradigm consisted of five alternating 30-s blocks of imagery and rest. Verbal cues (‘tennis,’ ‘navigation,’ or ‘relax’) indicated the onset of each condition, with ‘relax’ signalling rest periods during which participants were instructed to remain still with eyes closed. Univariate fMRI analysis was performed for all patients across both tasks using FSL software (https://fsl.fmrib.ox.ac.uk/fsl/fslwiki/); for each functional run, a general linear model (GLM) was specified, comprising alternating blocks of active imagery and rest.^66^ Statistical significance was determined at the cluster level using a threshold of *z* > 2.3 (cluster-corrected *P* < 0.05).^58,66^ In a subset of patients, significantly greater brain activation was found during either of the ‘tennis task’ (supplementary motor area) or ‘navigation task’ (parahippocampal place area) than rest, which was used as evidence for task-responsiveness.^58,66^ These patients were categorized (Table 1) as ‘fMRI+ positive task responders’ (labelled ‘positive’), whereas the rest were ‘fMRI- negative non-responders’ (labelled ‘negative’).

Resting-state fMRI data were collected over a 10-min acquisition period using a 3-tesla (3T) Siemens Trio scanner. Functional data comprised 300 EPI volumes with the following parameters: 33 slices; voxel size = 3 × 3 × 3.75 mm; TR = 2000ms; TE = 30 ms; flip angle = 78°. High-resolution anatomical T1-weighted images were obtained using a 3D sequence (1 mm isotropic voxels) with the following parameters: TR = 2300 ms; TE = 2.47 ms; 150 slices. Patients were excluded from further analysis if they met any of the following criteria: (i) large focal lesions exceeding one-third of a hemisphere or evidence of thalamic injury confirmed by neuroanatomical inspection; (ii) excessive motion of the head during resting-state acquisition (more than 3 mm translation and/or 3° rotation) or (iii) pre-processing failure i.e. segmentation and normalization.^26–28,60^

#### Data pre-processing

All functional and structural MRI data were pre-processed using SPM12 (https://www.fil.ion.ucl.ac.uk/spm/software/spm12/). The first five functional volumes were removed to allow for scanner equilibrium/steady-state magnetization. Slice-timing correction was performed on the fMRI volumes, followed by realignment to mean functional volume, which produced realignment parameters that were included in the first-level statistical models. Using the mean functional image, direct spatial normalization to EPI-template was conducted using the ‘old norm’ function in SPM due to reduced variability across subjects compared to other approaches in previous studies.^67^ The volunteers’ high-resolution structural images were co-registered to mean functional image or mean EPI (resulting from realignment stage) and segmented into grey matter, white matter, cerebrospinal fluid masks, and finally spatially normalized to the MNI-152 template.^68^ Visual inspection or quality control of images was conducted for normalization to standard space, which was essential for the DOC dataset due to the potential effect of lesions on spatial transformations. One subject was excluded due to the half of the brain missing from the volumes, and another subject was excluded due to complete failure of normalization. Denoising was then conducted in the CONN software (https://web.conn-toolbox.org),^69^ and functional images were smoothed at a FWHM Gaussian kernel of 6 mm. Movement parameters and their first temporal derivative were incorporated as first-level covariate to eliminate residual motion-related noise. Furthermore, the aCompCorr algorithm was applied to regressed out CSF, white-matter and motion-related signals from the time series (using first five principal components), which is a technique known to effectively remove movement-, respiratory- and cardiac-related artefacts,^70^ particularly in patients with disorders of consciousness,^71^ and has been validated for these kinds of datasets.^26,28,72^ Additionally, the Artefact Detection Tools pipeline (https://www.nitrc.org/projects/artefact_detect) in the CONN toolbox was also implemented to further remove motion-related artifacts in the time-series data by regressing out the effect of outlier scans (movement greater than 0.09 mm) in a first-level analysis; this procedure helps minimize localized motion artefacts not captured by the aCompCorr algorithm.^70,73^ Finally, linear detrending and a band-pass filter between 0.008 and 0.009 Hz were applied.

#### Thalamic parcellation atlas

We adopted seed-based functional connectivity (FC) analysis to establish which brain regions specific thalamic nuclei functionally interact with, where seeds (thalamic nuclei) need to be defined in regions of interest (ROIs).^69^ Although MRI offers superior visualization of soft biological tissue and enables the characterization of distinct structures, the intrinsic contrast of conventional T1- and T2-weighted MRI are insufficient to reliably distinguish individual thalamic nuclei, largely due to the relatively small volume of the thalamus (∼8 cm^3^/hemisphere), which highlights the need for developing thalamic parcellation methods. Existing digital atlases of the thalamus remain few in number and show limited cross-validation or anatomical correspondence with one another.^52^ A recently developed probabilistic atlas of thalamic subdivisions, derived from diffusion-weighted MRI data of 70 healthy Human Connectome Project volunteers, provides detailed mapping of thalamic organization.^53^ Diffusion-weighted MRI (DW-MRI) uniquely enables non-invasive characterization of white matter pathways within each thalamic nucleus in relation to their cortical projections. In this atlas^53^ the thalamus is segmented into seven regions closely matching the anatomical subparts, where six clusters correspond to histologically defined greater thalamic nuclei and the seventh cluster is a conglomerate encompassing three nuclei (Supplementary Fig. 1).^53^ This parcellation was selected as it provides an optimal balance between the spatial localization of thalamic voxels and their local diffusion characteristics, while showing strong correspondence with thalamic anatomy as defined in Morel’s stereotactic atlas.^53,74,75^ Atlas standard space of the thalamus probabilistic masks was transformed into the pre-processed standard space using nearest neighbour interpolation to avoid overlapping of thalamic masks and impact of results.^76^ Thalamic masks used correspond to (bilateral) nuclei: Pu, Ant, MD, VLD, central-lateral, lateral-posterior, medial-pulvinar group (CL-LP-MPu), VA and VLV (Supplementary Figs 2 and 3).

#### Statistical analysis

We analysed the fMRI data of *N* = 16 healthy controls under deep sedation (mean plasma propofol concentration of 2.68 *μ*g/ml) and *N* = 22 DOC patients. FC was calculated using CONN (https://web.conn-toolbox.org) using seed-to-voxel analyses from 7 seeded thalamic nuclei aiming to investigate thalamic nuclei interactions across the whole brain. Temporal correlations between each thalamic seed and all other brain voxels were computed using a GLM. For the anaesthesia dataset where the experimental design is within-subject, FC analyses were first conducted individually, then the individual seed-to-voxel parameter estimate images were entered into group-level analyses. Specifically, paired-sample *t* tests were used for differences in the participants among the conditions of deep sedation versus awake and recovery versus deep sedation. The cortical SPM-*t* maps from the individuals, both unthresholded and thresholded, were grouped together with a one-sample *t*-test. For the DOC dataset having a between-subject design, two-sample *t*-test (DOC versus control) was conducted at group-level. We compared the DOC group to the healthy awake participants (‘control’ group) from the anaesthesia experiment. The group-level inference of both datasets was corrected for ­multiple comparisons using random field theory, with a voxel-level threshold of *P* < 0.005 (uncorrected) and cluster level of *P* < 0.05 (family-wise error FWE-corrected for multiple comparisons).^77^

Radar plots are additionally presented to indicate the intrinsic connectivity network (ICN) spatial involvement (ICNi) in the contrasts of deep sedation versus awake and DOC versus control i.e. shows the voxel overlap between the FC results and canonical ICNs. These canonical ICNs were defined by an atlas^78^ containing 10 well-matched resting-state networks (RSN) from the *ICN_atlas* toolbox (https://www.nitrc.org/projects/icn_atlas/).^79^ Description of ICN-RSN atlas: VN 1, 2, and 3 (‘*visual network*’): medial, occipital pole, lateral visual areas; DMN (‘*default mode network*’): medial parietal (precuneus and posterior cingulate), bilateral inferior–lateral–parietal and ventromedial frontal cortex; CN (‘*cerebellum network*’): cerebellum; SMN (‘*sensorimotor network*’): supplementary motor area, sensorimotor cortex, secondary somatosensory cortex; AUDN (‘*auditory network*’): superior temporal gyrus, Heschl's gyrus, posterior insular, including primary and association auditory cortices; ECN (‘*executive control network*’): medial–frontal areas, such as anterior cingulate and paracingulate; FPN1 and 2 (‘*frontoparietal network*’): frontoparietal areas; which are the only maps to be strongly lateralized. In addition, FPN1 corresponds strongly to perception, somesthesis, and pain, and FPN2 to cognition and language paradigms, concomitant with Broca's and Wernicke's areas.^57^ Furthermore, to classify patterns of thalamo-cortical rs-FC findings based on network involvement (ICNi) with respect to individual nuclei, we performed hierarchical clustering using the *pheatmap* package in RStudio.^80^ We used Euclidean as the distance metric and complete linkage clustering for its sensitivity to outliers.

To investigate which nucleus had the greatest magnitude of change in FC with loss of consciousness, we computed in Matlab the average difference (unsigned magnitude) in FC (ΔFC) across the whole brain between the baseline (awake condition) and experimental comparison (deep sedation or DOC condition). Mean and standard deviations were used to normalize the values for ΔFC (z-scores). The subsequent statistical testing was computed in R and visualized in a box plot, where the y-axis represents the z-scored differential FC, and the x-axis depicts the specific thalamic nuclei. For statistical models, we used mixed linear model design wherever possible to correct for interpersonal variabilities (with the package ‘lme4’ in R). In these cases, we used the main effect as the fixed effect, and the subject ID as the random effect. The main effects which differ in different statistical tests were reported: for the DOC group comparison, the main effect for the ANOVA comparing the nucleus with the greatest magnitude of ΔFC with the rest of the nuclei was the difference of the dFC between this specific nucleus and the rest. The comparison for DOC subgroups did not have a within-individual structure, hence we did not conduct mixed linear models but used two-sample *t*-tests for that.

## Results

Our findings address, firstly, *which regions—or nuclei—*of the thalamus were most strongly associated with loss of consciousness in both anaesthesia and DOC, and secondly, *what mechanisms—or cortical networks—*accounted for the functional changes between the respective thalamic nuclei and the rest of the brain. We thus present a whole-brain analysis of thalamo-cortical (and sub-cortical) FC changes across pharmacological and pathological loss of consciousness. We analysed the fMRI data of *N* = 16 healthy controls under deep sedation (mean plasma propofol concentration of 2.68 *μ*g/ml) and *N* = 22 DOC patients (*N* = 13 in MCS and *N* = 9 in UWS). We used a thalamic parcellation atlas based on DW-MRI, where the thalamus is segmented into 7 ROIs closely matching the anatomical subparts in Morel’s atlas.^53^

### The pulvinar nuclei demonstrated the strongest cortical functional connectivity change in anaesthetic-induced loss of consciousness in healthy subjects

**Figure 1:**
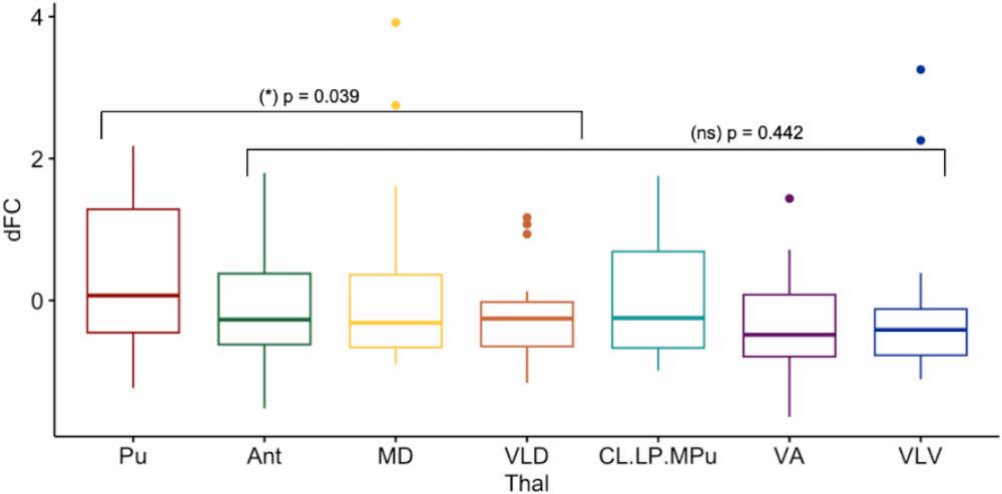
**Magnitude of change in functional connectivity (ΔFC) in anaesthesia group comparison.** Box plot displays z-scores for the anaesthesia group comparison (*n* = 16), where among all nuclei, the Pu had the greatest magnitude of ΔFC in anaesthesia. For the statistical model, we used mixed linear model design to correct for interpersonal variabilities (with the package ‘lme4’ in R), with the main effect as the fixed effect and the subject ID as the random effect, to compare the magnitude of change in FC. Indeed, under anaesthesia, the ΔFC for Pu was found to be statistically significant in comparison to the rest of the nuclei (*t* = 2.08, *P* = 0.039*). The rest of the nuclei were found to not be significantly different from each other in anaesthesia (*P* = 0.442) with an *F*-test (ANOVA).

First and foremost, we sought to investigate which thalamic nucleus was most associated with the overall FC changes in the brain with pharmacological (anaesthetic-induced) loss of consciousness. Thus, to determine which nucleus had the greatest magnitude of change in FC with loss of consciousness, we computed the average change or differential FC (ΔFC) across the whole brain between the baseline (FC maps indexed by individual’s correlation coefficient *Beta* for ‘awake condition’) and experimental comparison (FC maps for ‘deep sedation condition’). Mean and standard deviations were used to normalize the values for ΔFC (z-scores) displayed in a box plot where the y-axis represents the z-scored ΔFC and the x-axis depicts the specific thalamic nuclei (Fig. 1). Among all nuclei, the Pu was found to have the greatest magnitude of ΔFC in healthy subjects with pharmacological loss of consciousness. We then tested, based on our preliminary finding, whether the Pu had the strongest ΔFC—significantly greater change—than the ΔFC of the rest of the nuclei. Thus, a linear mixed model correcting for individual differences was implemented to test for the significance of the Pu connectivity. We compared Pu group against the group of all other nuclei, and indeed, under anaesthesia, the ΔFC for Pu was found to be significant in comparison to the rest of the nuclei (*t* = 2.081, *P* = 0.039). [All other nuclei were found to not be significantly different from each other in anaesthesia (*P* = 0.442) following an *F*-test (ANOVA)].

### Functional interactions between individual thalamic nuclei and whole brain in healthy volunteers under deep anaesthesia

**Figure 2:**
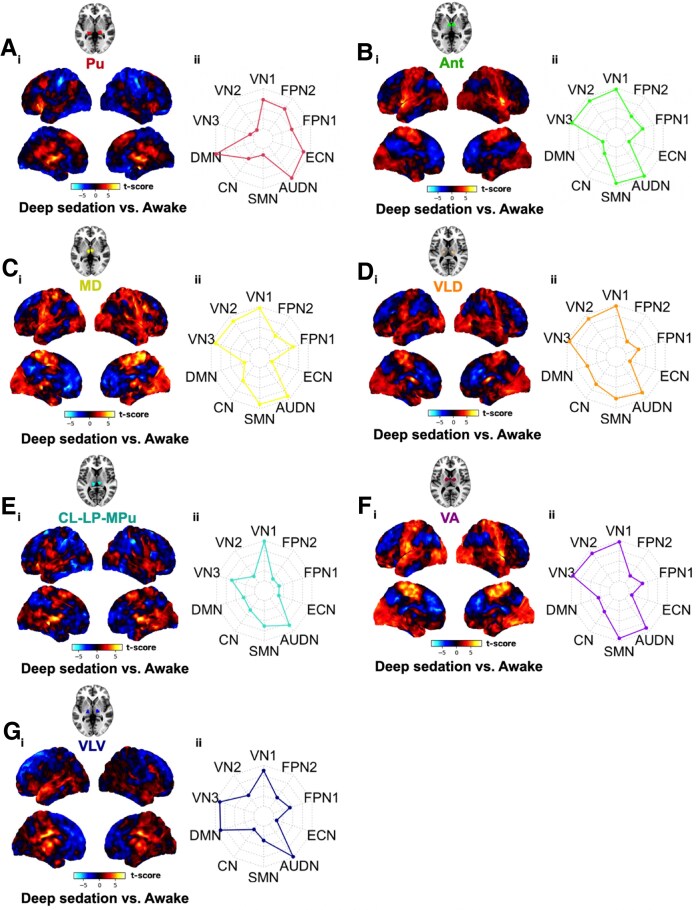
**Whole-brain resting-state (rs) functional connectivity (FC) maps in heathy volunteers in pharmacological (propofol-induced) loss of consciousness.** Temporal correlations for thalamic seeds were computed for all other voxels in the brain using a GLM. For the anaesthesia dataset (*n* = 16) where the experimental design is within-subject, FC analyses were first conducted individually, then individual seed-to-voxel parameter estimate images were entered into group-level analysis. Specifically, paired-sample *t* tests were used for differences in the participants among the condition of deep sedation versus awake. The cortical SPM-*t* maps from the individuals, both unthresholded and thresholded, were grouped together with a one-sample *t*-test. The group-level inference of the dataset was corrected for ­multiple comparisons using random field theory [voxel level threshold of *P* < 0.005 (uncorrected) and cluster level *P* < 0.05 (FWE-corrected for multiple comparisons)].^77^ Results were computed from seed-to-voxel rs-FC analysis across 7 respective thalamic nuclei [bilateral] seeds — Pu, Ant, MD, VLD, CL-LP-MPu, VA, VLV — and the rest of the brain. Colour bar denotes the strength of the t-statistic. Unthresholded t-maps of the deep sedation versus awake contrast are shown (**Ai, Bi, Ci, Di, Ei, Fi, Gi**). Radar plots indicate the intrinsic connectivity network (ICN) spatial involvement (ICNi) of brain regions in the deep sedation versus awake contrast i.e. shows the voxel overlap between the FC results and canonical ICNs (**Aii, Bii, Cii, Dii, Eii, Fii, Gii**). These canonical ICNs were defined by an atlas^78^ containing 10 well-matched resting-state networks (RSN) from the *ICN_atlas* toolbox (https://www.nitrc.org/projects/icn_atlas/).^79^ Description of ICN-RSN atlas: VN 1, 2, and 3 (‘*visual network*’): medial, occipital pole, and lateral visual areas; DMN (‘*default mode network*’): medial parietal (precuneus and posterior cingulate), bilateral inferior–lateral–parietal, and ventromedial frontal cortex; CN (‘*cerebellum network*’): cerebellum; SMN (‘*sensorimotor network*’): supplementary motor area, sensorimotor cortex, and secondary somatosensory cortex; AUDN (‘*auditory network*’): superior temporal gyrus, Heschl's gyrus, and posterior insular. It includes primary and association auditory cortices; ECN (‘*executive control network*’): medial–frontal areas, including anterior cingulate and paracingulate; FPN1 and 2 (‘*frontoparietal network*’): frontoparietal areas; these are the only maps to be strongly lateralized. In addition, FPN1 corresponds strongly to perception–somesthesis–pain, and FPN2 to cognition–language paradigms, consistent with Broca's and Wernicke's areas.^57^

We then investigated the spatial localization of FC changes between thalamic nuclei and the rest of the brain with anaesthetic-induced loss of consciousness in healthy volunteers. We computed whole-brain connectivity (normalized as SPM-t) maps following seed-based FC analysis (contrast of deep sedation versus awake). Visually, SPM-t maps revealed that there were differential FC or cortical network patterns of connectivity that correspond to the discrete thalamic nuclei (Fig. 2Ai–Gi), following pharmacological loss of consciousness. Correspondingly, these FC patterns were reversed with recovery of consciousness from anaesthesia (Supplementary Fig. 4A–G), validating our findings.

**Figure 3:**
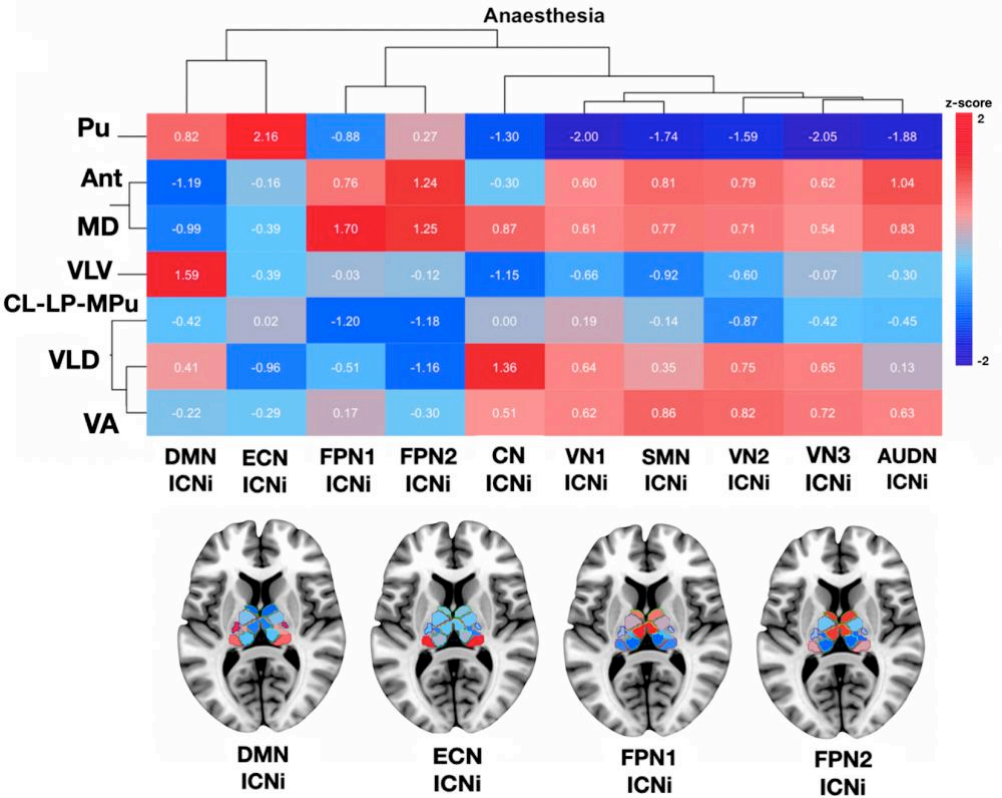
**Hierarchical clustering of thalamo-cortical resting-state (rs) functional connectivity (FC) in heathy volunteers in pharmacological (propofol-induced) loss of consciousness.** To classify patterns of thalamo-cortical rs-FC findings based on network involvement (ICNi) with respect to individual nuclei, we performed hierarchical clustering using the pheatmap package in RStudio.^80^ We used Euclidean as the distance metric and complete linkage clustering for its sensitivity to outliers. The algorithm computed clusters based on thalamic nuclei (y-axis), where Pu and VLV were grouped into two separate clusters; the Ant and MD were grouped into a third cluster; and the CL-LP-MPu, VLD, and VA were grouped into a fourth cluster. Additionally, the algorithm computed clusters based on cortical networks (x-axis), and found that the higher-order networks were grouped separately (i.e. FPN1 and FPN2 into one cluster, and DMN and ECN into a separate cluster) from the lower-order networks (i.e. all other networks, including SM, VN, AUDN, and CB, into additional clusters).

Then, to quantify more precisely the cortical network involvement in the SPM-t maps, we overlapped our maps with ICNs and calculated the ICN involvement with a resting-state brain network atlas^78^ using the *ICN_atlas* toolbox.^79^ We were able to further validate numerically that, indeed, there was prominent cortical network involvement that varied across the different thalamic nuclei (Fig. 2Aii–Gii). Finally, we used hierarchical clustering to identify these distinctive patterns of functional connectivity we found for each thalamic nucleus (Fig. 3). This complemented our visual and quantitative assessments, as it was evident there were commonalities but also differential functional connectivity profiles across thalamic nuclei. With anaesthetic-induced loss of consciousness, the Pu and VLV were grouped into two separate clusters; the Ant and MD were grouped into a third cluster; and the CL-LP-MPu, VLD and VA were grouped into a fourth cluster. Additionally, the algorithm computed clusters based on cortical networks, and found that the higher-order networks were grouped separately (i.e. FPN1 and FPN2 into one cluster, and DMN and ECN into a separate cluster) from the lower-order networks (i.e. all other networks, including SM, VN, AUDN and CB, into additional clusters). Thus, as a follow-up to our previous findings (Fig. 1), we identified that the Pu demonstrated strong FC changes with the DMN, accompanied by even stronger FC changes with the ECN, providing substantiation to the proposition that the Pu may manifest more acute contributions than other nuclei in anaesthetic-induced loss of consciousness, through interactions with the higher-order DMN and ECN networks. (With all other cortical networks—which were exclusively lower order networks except for FPN1—the Pu revealed opposite FC changes). Remarkably, therefore, the nucleus with the strongest delta FC (quantitative change) was the same one that also demonstrated the most distinct spatial pattern of FC changes across the cortex (qualitative change).

### The ventral-latero-ventral nuclei exerted the strongest cortical functional connectivity change in pathological loss of consciousness in disorders of consciousness patients

**Figure 4:**
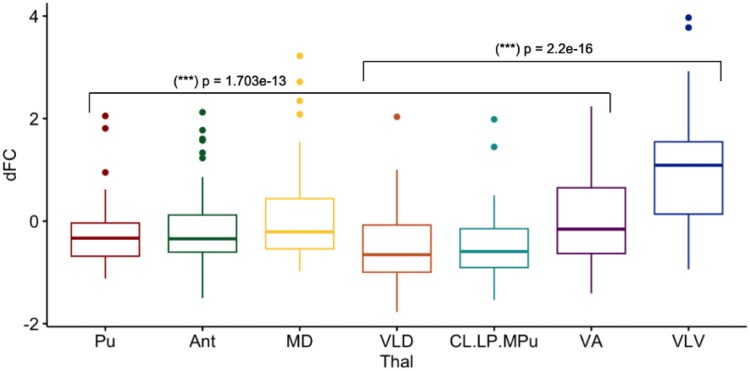
**Magnitude of change in functional connectivity (ΔFC) in DOC group comparison.** Box plot displays z-scores for the DOC group comparison (*n* = 22), where among all nuclei, the VLV had the greatest magnitude of ΔFC in DOC. Linear mixed models for within-subject analysis were used to compare the magnitude of change in FC. The ΔFC for VLV was found to be statistically significant in comparison to the rest of the nuclei (t = 12.336, *P* = 2.2e-16***) with an F-test (ANOVA). Owing to significant differences found among the rest of the nuclei in DOC (*P* = 1.703e-13), we made multiple comparisons between VLV with each of the rest of the nuclei (where the main effect for the ANOVA comparing VLV with the rest of the nuclei was the difference of the dFC between the VLV and the rest of the nuclei.) After multiple comparison, VLV was significantly the highest among all.

Analogous to our previous approach, we sought to identify which thalamic nucleus was most associated with the overall FC changes with pathological (DOC-induced) loss of consciousness. Thus, to determine which nucleus had the greatest magnitude of change in FC with loss of consciousness, we computed the average difference in FC (ΔFC) across the whole-brain between the baseline (‘awake condition’) and experimental comparison (‘DOC condition’). Mean and standard deviations were used to normalize the values for ΔFC (z-scores) displayed in a box plot where the y-axis represents the z-scored ΔFC and the x-axis depicts the specific thalamic nuclei (Fig. 4). Among all thalamic nuclei, VLV was found to have the greatest magnitude of ΔFC with pathological loss of consciousness. We then wanted to test whether our preliminary finding that the VLV had the strongest ΔFC is statistically more significant than the ΔFC of the rest of the nuclei. Hence, a linear mixed model correcting for individual differences was implemented to test for the significance of the VLV connectivity. Indeed, in DOC, the ΔFC for VLV was found to be statistically significant in comparison to the rest of the nuclei (*t* = 12.336, *P* = 2.2e-16***) with an *F*-test (ANOVA). Owing to significant differences found among the rest of the nuclei in DOC (*P* = 1.703e-13), we made multiple comparisons between VLV with each of the rest of the nuclei. After correcting significance level for the multiple comparisons, VLV was shown significantly the highest among all. We suggest that the generality of significant alterations in the thalamic FC was partly due to the between-subject design of the dataset where the DOC group has considerable individual variations owing to various aetiologies, including TBI, global hypoxic-ischaemic encephalopathy, ischaemic stroke and other causes.^7,62^

### Functional interactions between individual thalamic nuclei and whole brain in disorders of consciousness patients

**Figure 5:**
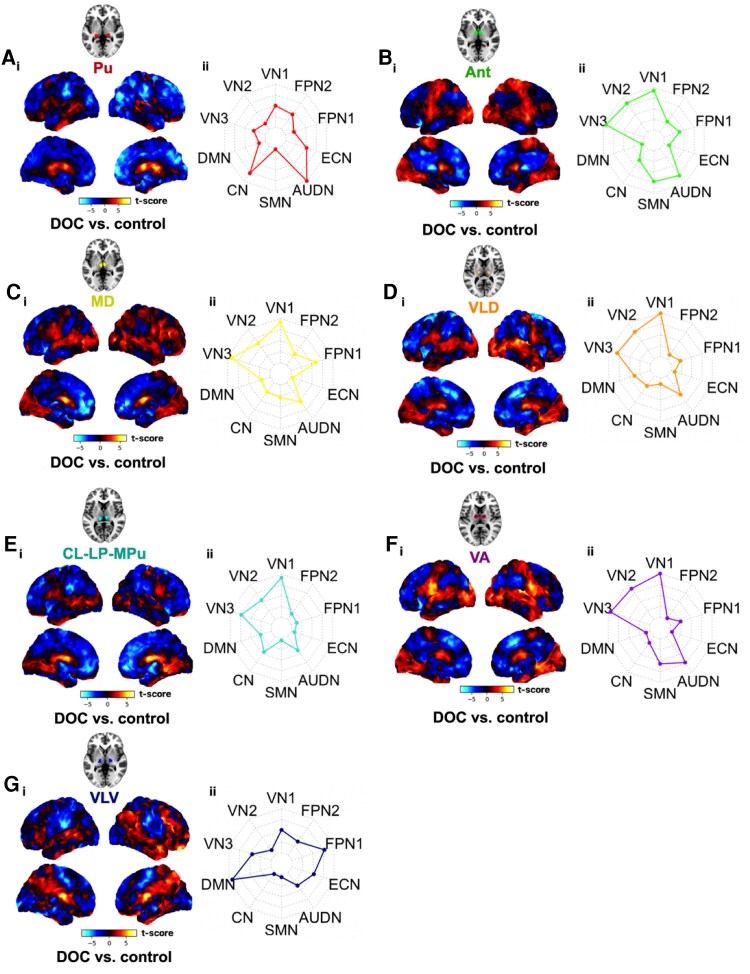
**Whole-brain resting-state (rs) functional connectivity (FC) maps in DOC patients in loss of consciousness.** Temporal correlations for thalamic seeds were computed for all other voxels in the brain using a GLM. For the DOC dataset (*n* = 22) having a between-subject design, two-sample *t*-test (DOC versus control) was conducted at the group level. The group-level inference of the dataset was corrected for ­multiple comparisons using random field theory [voxel level threshold of *P* < 0.005 (uncorrected) and cluster level *P* < 0.05 (FWE-corrected for multiple comparisons)].^77^ Results were computed from seed-to-voxel rs-FC analysis across 7 respective thalamic nuclei [bilateral] seeds—Pu, Ant, MD, VLD, CL-LP-MPu, VA, VLV—and rest of the brain. Colour bar denotes the strength of the t-statistic. Unthresholded t-maps of the DOC versus control contrast are shown (**Ai, Bi, Ci, Di, Ei, Fi, Gi)**. Radar plots indicate the intrinsic connectivity network (ICN) spatial involvement (ICNi) of brain regions in the DOC versus control contrast i.e. shows the voxel overlap between the FC results and canonical ICNs (**Aii, Bii, Cii, Dii, Eii, Fii, Gii**). These canonical ICNs were defined by an atlas^78^ containing 10 well-matched resting-state networks (RSN) from the *ICN_atlas* toolbox (https://www.nitrc.org/projects/icn_atlas/).^79^ Description of ICN-RSN atlas: VN 1, 2, and 3 (‘*visual network*’): medial, occipital pole, and lateral visual areas; DMN (‘*default mode network*’): medial parietal (precuneus and posterior cingulate), bilateral inferior–lateral–parietal, and ventromedial frontal cortex; CN (‘*cerebellum network*’): cerebellum; SMN (‘*sensorimotor network*’): supplementary motor area, sensorimotor cortex, and secondary somatosensory cortex; AUDN (‘*auditory network*’): superior temporal gyrus, Heschl's gyrus, and posterior insular. It includes primary and association auditory cortices; ECN (‘*executive control network*’): medial–frontal areas, including anterior cingulate and paracingulate; FPN1 and 2 (‘*frontoparietal network*’): frontoparietal areas; these are the only maps to be strongly lateralized. In addition, FPN1 corresponds strongly to perception–somesthesis–pain, and FPN2 to cognition–language paradigms, consistent with Broca's and Wernicke's areas.^57^

We then explored the spatial localization of FC changes between thalamic nuclei and the rest of the brain with pathological loss of consciousness in DOC patients. We computed whole-brain connectivity (normalized as SPM-t) maps following seed-based FC analysis (contrast of DOC versus control). Visually, SPM-t maps reveal that there were differential FC or cortical network patterns of connectivity that correspond to the discrete thalamic nuclei (Fig. 5Ai–Gi), with pathological loss of consciousness.

**Figure 6:**
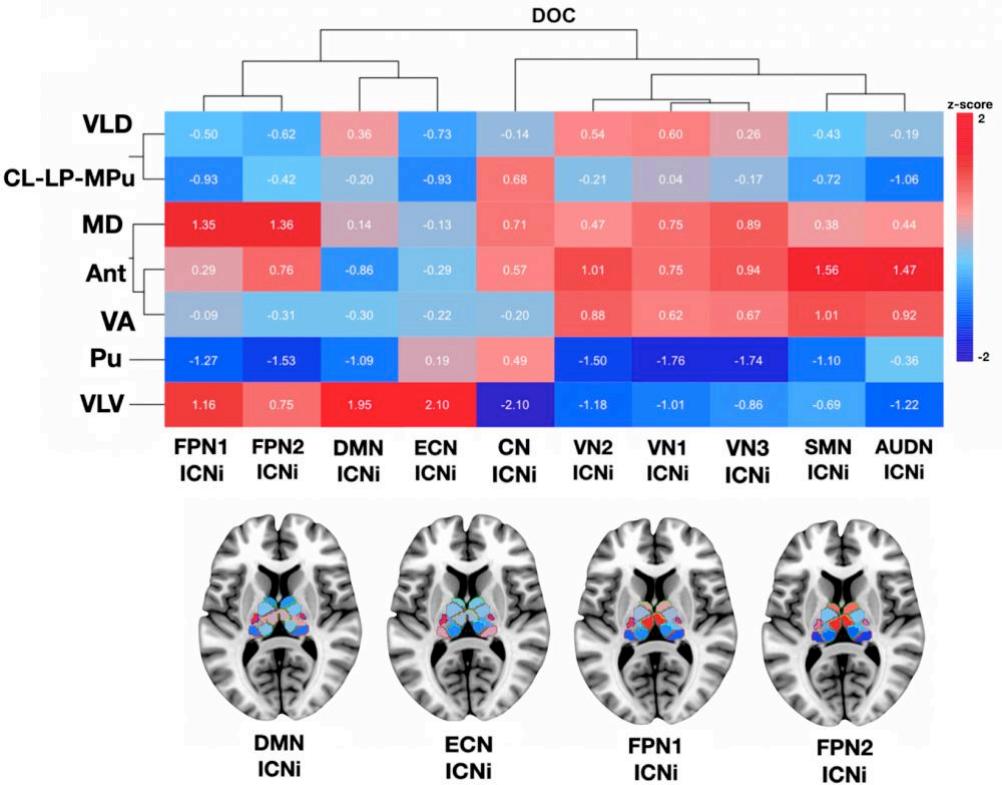
**Hierarchical clustering of thalamo-cortical resting-state (rs) functional connectivity (FC) in DOC patients in loss of consciousness.** To classify patterns of thalamo-cortical rs-FC findings based on network involvement (ICNi) with respect to individual nuclei, we performed hierarchical clustering using the pheatmap package in RStudio.^80^ We used Euclidean as the distance metric and complete linkage clustering for its sensitivity to outliers. The algorithm computed clusters based on thalamic nuclei (y-axis), where the VLD and CL-LP-MPu were grouped into one cluster; the MD, Ant, and VA were grouped into a second cluster; and the Pu and VLV were grouped into a third and fourth cluster respectively. Furthermore, the algorithm computed clusters based on cortical networks (x-axis), and found that the higher-order networks were grouped separately (i.e. FPN1 and FPN2 into one cluster, and DMN and ECN into a separate cluster) from the lower-order networks (i.e. all other networks, including SM, VN, AUDN, and CB, into additional clusters).

Next, to quantify the cortical network involvement in the SPM-t maps, we overlapped our maps with ICNs and calculated the ICN involvement with a resting-state brain network atlas^78^ using the *ICN_atlas* toolbox.^79^ We were able to further validate numerically that, indeed, there was prominent cortical network involvement that varied across the different thalamic nuclei (Fig. 5Aii–Gii). Finally, we used hierarchical clustering to identify these distinctive patterns of functional connectivity we found for each thalamic nucleus (Fig. 6). This complemented our visual and quantitative assessments, as it was evident there were commonalities but also differential functional connectivity profiles across thalamic nuclei. With loss of consciousness in DOC, the VLD and CL-LP-MPu were grouped into one cluster; the MD, Ant and VA were grouped into a second cluster and the Pu and VLV were grouped into a third and fourth cluster respectively. Furthermore, the algorithm computed clusters based on cortical networks, and found that the higher-order networks were grouped separately (i.e. FPN1 and FPN2 into one cluster, and DMN and ECN into a separate cluster) from the lower-order networks (i.e. all other networks, including SM, VN, AUDN and CB, into additional clusters). Hence, as a follow-up to our previous findings presented earlier in Fig. 4, we identified that the VLV demonstrated strong FC changes with *all* higher-order networks, including the FPN1, FPN2, DMN and ECN. (With all other cortical networks—which were solely lower-order networks—the VLV revealed opposite FC changes). Crucially, therefore, the nucleus with the strongest delta FC (quantitative change) was the same one that also demonstrated the most distinct spatial pattern of FC changes across the cortex (qualitative change). This consolidates our premise that the VLV, through interactions with higher-order networks, may play a more pivotal role in pathological perturbations of consciousness than previously explored. Our findings complement previous lines of evidence highlighting the role of VLV as an integrative centre for motor control, receiving cortical-cerebellar-basal ganglia input and sending core-like projections to primary motor areas.^81,82^

### Behavioural correspondence with the thalamic functional connectivity changes

**Figure 7:**
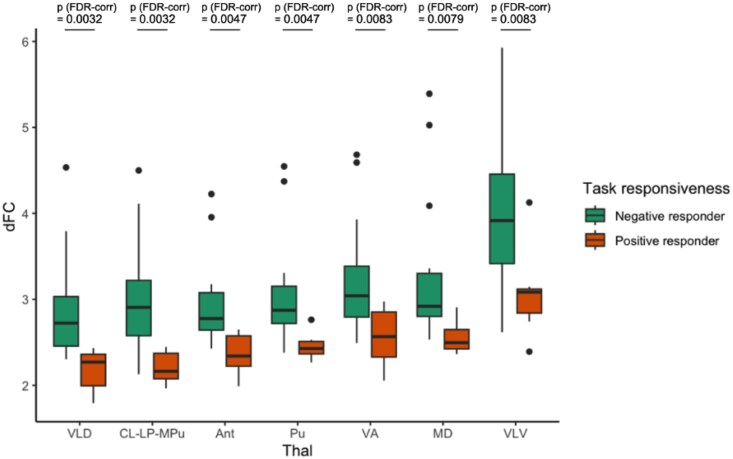
**Magnitude of change in functional connectivity (ΔFC) in DOC subgroup comparison.** Box plot displays ΔFC scores corresponding to each DOC subgroup (i. fMRI-/task non-responders and ii. fMRI+ /task responders) (*n* = 22) differential FC from healthy controls. For statistical models, as this comparison does not have a within-individual structure, we used two-sample t-tests. Among all nuclei, the VLV had the greatest ΔFC between task non-responders and task responders after FDR correction.

In our final analysis (Fig. 7), we sought to further establish thalamic changes in the DOC patients by subjecting them to a test, which assessed their levels of covert consciousness. This test involved tasks of motor imagery (‘tennis task’) and spatial imagery (‘navigation task’), which allowed us to split DOC patients into two categories: those with positive responses to the task (‘fMRI+ or task responders’) and those with negative responses to the task (‘fMRI- or task non-responders’). All positive task-responders (*n* = 7) were TBI patients (except for one patient) and were exclusively MCS patients. Among those who did not respond to the task (*n* = 15), six patients were MCS and nine patients were UWS. We used this test as it has been demonstrated to be a more reliable proxy for consciousness because it provides a more sensitive evaluation of the cognitive functions of DOC patients that could not be readily observable with standard behavioural diagnosis such as MCS and UWS.^64,65,83,84^ This is especially vital given the high rate of misdiagnosis in DOC patients.^85,86^ Concretely, this implies that the subgroup of positive task responders are less severely impaired on the DOC continuum, whereas the negative non-responders consists of more severe patients.

We proceeded with testing the behavioural relevance of FC alterations found before by investigating the differential FC (ΔFC) of thalamic nuclei between each distinct subgroup of DOC patients and healthy controls. The subgroups include those with negative fMRI responses to the Tennis Task (‘task non-responders’, fMRI-) and those with positive fMRI responses (‘task responders’, fMRI+). Across all 7 thalamic nuclei, the ΔFC was found to be statistically significant following a two-sample *t*-test between the fMRI+ and fMRI- subgroups, corrected for multiple comparisons (FDR-correction). In line with our previous findings (Fig. 4), the greatest ΔFC across the two subgroups was found for the VLV (*P* = 0.0083). Crucially, the VLV is significantly responsible for FC changes in pathological loss of consciousness overall, and it is even more prominent in the cohort of more severely impaired DOC patients (negative non-responders). This result reinforces our previous analysis and suggests the utility of VLV, an integral part of the motor thalamus, as not merely a reliable neural marker for consciousness but also, crucially, a behavioural marker that is specific to patients with DOC, particularly those at the more severe end of the spectrum.

## Discussion

We sought to identify the contributions of major subdivisions of the thalamus to perturbed states of consciousness in pharmacological (propofol-induced anaesthesia) and pathological (DOC) models in humans. Using fMRI, we aimed to explore multiple distinct nuclei of the thalamus in loss of consciousness by revealing whole-brain FC changes. Specifically, we examined not only where the largest in magnitude, thalamo-cortical FC alterations occurred but also in which specific thalamic nucleus these changes were most prominent. We found that, among all thalamic nuclei, the Pu was found to have the biggest magnitude of change or strongest differential FC (ΔFC) with anaesthetic-induced loss of consciousness in healthy volunteers, which demonstrated strong FC changes with higher-order ECN and DMN and weaker FC changes with FPN2 cortical networks (and opposite FC changes with all other cortical networks, particularly lower-order). Remarkably, by contrast, in the patient cohort where loss of consciousness was induced by pathological reasons (i.e. DOC), the VLV was found to have the strongest ΔFC among thalamic nuclei, which demonstrated strong FC changes with *all* higher-order cortical networks, including the FPN1, FPN2, DMN and ECN (whilst revealed opposite FC changes with all other, exclusively lower-order, cortical networks). Crucially, we highlight that this finding is not only a prominent neural marker but also reflects changes at the behavioural level, which evidently has important clinical implications.

Our findings of the Pu demonstrating the strongest impact on overall ΔFC in anaesthetic-induced loss of consciousness are in line with previous work demonstrating that particularly the medial pulvinar region is involved not only in visuomotor and auditory processing but also associative and higher cognitive processing, and even more so, abnormal medial pulvinar connectivity was associated to neurodevelopmental disorders.^87^ The medial pulvinar was shown to be involved in altered states of consciousness, notably focal seizures,^88^ and, more recently, stimulation of this region led to improvements in awareness in pharmaco-resistant temporal lobe epilepsy.^89^ Furthermore, studies demonstrated that pulvinar lesions led to changes in conscious content—impaired feature binding^90^ and hemineglect^91^—in humans. On the other hand, in the DOC patient cohort, the VLV was found to have the strongest (ΔFC) among all nuclei in pathological loss of consciousness. The VLV, known to be part of the motor thalamus, is involved in action control in a circuitry with basal ganglia, motor, premotor and prefrontal cortices.^57,82,92^ More specifically, VL nuclei have connections to the globus pallidum internal (GPi) of the basal ganglia,^22,93^ and atrophy in this area has been associated with decreased behavioural arousal patients with DOC.^22^ Additionally, the VL has been implicated in higher-level cognition, particularly in the integration of attention and memory, has been demonstrated by evidence of VL lesions leading to impairments in stroke patients.^94^

Importantly, the involvement of distinct cortical networks may account for the strongest whole-brain ΔFC identified for the Pu in pharmacological and VLV in pathological loss of consciousness. Higher-order networks exhibited strong FC changes with the Pu and VLV, in the healthy anaesthetized and DOC patient cohorts, respectively. In both types of loss of consciousness, the DMN and ECN, considered to be core consciousness hubs, were highly involved, though stemming from *different* thalamic hubs. Importantly, the strongest FC changes for the VLV were demonstrated with the ECN. Additionally, other higher-order networks, i.e. FPN were found to have the strongest FC changes with VLV in pathological loss of consciousness, confirming the importance of such consciousness-related networks. Thus, there may be shared mechanisms involving cortical network interactions in pharmacological and pathological loss of consciousness, though they have associations with different thalamic loci.

Our results confirm previous literature surrounding the importance of large-scale consciousness-supporting networks, both in health and disease.^4–6,8–11,95^ Among these, the default mode network, DMN, well-known to be largely linked to conscious states,^5,96–98^ is a widely distributed network consisting of medial frontal and posterior medial parietal cortices, angular gyrus and hippocampus, of which the posteromedial cortex is a major connectivity hub.^99^ It has been increasingly associated with mediating internal (self-related) and external (environmental) information processing.^98,100^ The posteromedial area has been demonstrated to play a key role in altered consciousness in epileptic patients^101–103^ and in patients with disorders of consciousness.^28,104,105^ Converging evidence has shown the importance of cortical and subcortical contributions of the DMN, and especially posteromedial areas, in healthy subjects under anaesthesia and/or patients with DOC.^7,27,28^ Our results align with several resting-state fMRI studies in healthy volunteers which have demonstrated strong FC between precuneus and whole thalamus during changes in consciousness.^106,107^ Importantly, specific thalamic nuclei were found to be both functionally and structurally connected to nodes of the default mode network.^107–111^ Using tractography analyses of diffusion MRI data, Edlow and colleagues mapped projection pathways connecting brainstem default ascending arousal network nodes with thalamic—intralaminar, reticular, and paraventricular nuclei—and cortical DMN nodes in healthy controls.^112^ Furthermore, past work has shown that alterations in structural connectivity in DMN and along the thalamus-precuneus pathway were correlated with behavioural signs in patients with impaired consciousness,^63^ reinforcing the importance of posteromedial regions in the neural basis of consciousness. Evidence of compromised connectivity between the precuneus and whole thalamus associated with impaired consciousness have been further demonstrated in various MCS and UWS cohorts.^113–115^ Additionally, PET evidence indicates that UWS patients display altered effective connectivity between the posterior cingulate and frontal association regions,^116^ as well as between the anterior cingulate cortex, prefrontal cortex, and thalamus.^107^ Moreover, network property alterations and FC disruptions were found in brain regions associated with consciousness, particularly the medial parietal cortex, frontal cortex and thalamus in DOC patients.^117^ Furthermore, a functional regulation of the DMN by the thalamus has been suggested in other impaired states of consciousness, specifically during epileptic seizures.

Among other higher-order cortical networks relevant to our findings, the executive control (ECN) network, which consists of dorsolateral frontal and parietal nodes, is known for mediation of attention and awareness of one’s environment.^103,118–120^ There is also increased evidence of its alterations in disorders of consciousness, where fewer MCS and UWS patients following brain injury showed components of neuronal origin for the left and right ECN, as well as for DMN and auditory networks, compared to healthy controls.^15^ Furthermore, dynamic interactions between the ECN and salience network have been found important for sustaining and recovering consciousness.^14^ Similarly, the FPN is associated with restoring cerebral activity in recovery from disorders of consciousness,^114,121^ and is known to have robust connectivity to subcortical and limbic structures, notably the thalamus and basal ganglia.^20,95,113^ Finally, our results demonstrated strong FC changes with higher-order networks (i.e. DMN, ECN, FPN) and opposite FC changes with lower-order networks (i.e. SMN, VN, AUDN, CBN) in our DOC patient cohort. It remains to be explored precisely how this functional network antagonism may play a role in sustaining consciousness in larger cohorts of patients.^122^ Clearly, our findings demonstrate that distinct cortical networks interact with different thalamic nuclei depending on the nature of loss of consciousness, whether pharmacological or pathological, thus opening an interesting avenue for further inquiries into the importance of thalamo-cortical aka ‘*thalamic nuclei-cortical network’* interactions which seem to contribute to the phenomena of loss of consciousness.

On a more microscopic level, two distinct cell classes in the thalamus have been shown to be differentially correlated with the cortex, which includes parvalbumin-staining (PVALB) core cells driving excitatory activity via projections to cortical layers III and IV) and calbindin-staining (CALB1) matrix cells projecting diffusely to supra-granular and infragranular layers.^17,33,123^ Intriguingly, previous work showed that CALB1-rich matrix cells in the healthy awake brain exhibited strong preferential functional coupling with several cortical networks, involving the control, limbic, ventral attention, and notably the DMN, whereas in contrast, PVALB-rich core cells favoured functional coupling with visual, dorsal-attention, temporo-parietal and somatomotor networks.^124^ Moreover, Huang and colleagues observed a reorganization from a balanced core-matrix unimodal-transmodal functional geometry during consciousness to unimodal core dominance during loss of consciousness with propofol in humans.^125^ Additionally, parvalbumin expression was found to be higher in areas with reduced cortical connectivity during sedation with propofol.^126^ There remains a need to further investigate the coupling effects between cortical networks and matrix or core cells in thalamic nuclei in the anaesthetized brain, and even more so, in patients with DOC.

Owing to recent advancements in thalamic parcellation,^51,52^ our study has demonstrated that specific sections of the thalamus are associated with distinct types of perturbations of consciousness, pathological^7^ or pharmacological.^127^ Previously, the thalamus had been explored almost exclusively as a whole entity, and few studies have been able to break down the heterogenous complex nuclei and explore their respective relevance in consciousness in humans.^42–44^ In a recent animal study led by Tasserie and colleagues, electrical stimulation of the ‘central thalamus’, notably CM nuclei, was found to restore arousal and awareness in a macaque model in propofol-induced loss of consciousness.^40^ Although stimulation of the ventral thalamus control site had no effects on behavioural score in macaques, the authors acknowledged there was no data acquired during an event-related auditory task experiment to measure conscious awareness following ventral lateral thalamic stimulation under anaesthesia,^40^ pressing the need for more extended and nuanced investigations. Furthermore, Bastos and colleagues^128^ implanted multiple-contact stimulating electrodes in frontal thalamic nuclei (i.e. intralaminar, mediodorsal, with additional targets in neighbouring ventral posterolateral nucleus) in macaques, and demonstrated that thalamic stimulation reversed the electrophysiologic patterns associated with unconsciousness.

Given our findings, not only do we provide a differential picture between pharmacological and pathological states of unconsciousness with relevance to the thalamus but we also press for the need for more nuanced experimental paradigms that distinguish arousal (alertness or wakefulness) from awareness (content of subjective experience) in human consciousness.^129–131^ One model specific to aetiologies of brain injuries producing DOC has been proposed in the clinical literature—the ‘mesocircuit model’—,which suggests a downregulation of synaptic activity across areas of the frontal cortex (i.e. medial frontal, anterior cingulate cortex), central thalamus (i.e. central-lateral nucleus) and striatum.^7,132^ Moreover, a functional disfacilitation of the central thalamus, following injuries, reduces activity along projections from central thalamus to the frontal cortex, posterior medial parietal cortex (a key DMN node) and striatum.^23^ Fridman and Schiff^23^ have previously outlined that the term ‘central thalamus’ refers to a functional and physiological construct rather than a strictly delineated anatomical entity.^23,133–135^ Furthermore, the targets for stimulation in 5 individual patients with impaired consciousness following traumatic brain injury were functional targets of the central-lateral/medial dorsal tegmental fibre bundle activations,^46^ modelled upon DTI data,^46,55,57^ though none of these patients were DOC. In our cohort of DOC patients, we provide evidence for a more complex picture in pathological disruptions of consciousness with an important role for the VLV, part of the motor thalamus, possibly through inputs from the globus pallidus and striatum from the basal ganglia circuitry.^16,82,93^ Moreover, it may be important to consider the proximity of ventral lateral ventral to central-lateral nuclei, as well as their relevant projections. Specifically, ventral lateral nuclei have projections to the globus pallidum internal of the basal ganglia,^110^ and atrophy in this area has been associated with decreased behavioural arousal patients with DOC.^104^ Alternatively, the central-lateral neurons innervate the rostral striatum of the basal ganglia, as well as prefrontal/frontal cortex; these CL projections drive action potentials from primary striatal output neurons, which, consequently, increase the inhibition of the globus pallidus interna, and release further thalamic activation of the cortical regions.^46^ We suggest, therefore, a potential interactive framework by which specific functional nodes—CL and VLV included—of the thalamus coordinate with the basal ganglia to modulate arousal in humans, calling for further experimental investigations.

Opening the ‘black box’ of the thalamus significantly contributes to unravelling the ‘black box’ of consciousness. By disentangling the thalamus into more nuanced anatomically relevant parts, we suggest that pharmacological and pathological loss of consciousness—although they overlap at the cortical^5,28,41,60,136^ and brainstem^27^ levels—appear to involve distinct thalamic contributions, given that we found the Pu in anaesthesia and VLV in DOC, respectively, to be pivotal. This has clinical implications, as DOC therapies may not be fully reliant upon anaesthetic studies, at least where thalamic connectivity is concerned. Unlike anaesthesia, which taps into the brain connectivity changes through transient neuromodulatory influences, the permanent anatomical damages that accompany brain injury may account for the differential thalamic involvement.^7,137^ By having further separated the DOC in two groups, those severely injured and those less impaired, and by having shown that the VLV remains the predominant nucleus responsible for both, we show consistency within the DOC category of loss of consciousness. Hence, our results not only demonstrate there is heterogeneity between consciousness alterations of anaesthesia and DOC in terms of thalamic contributions but also homogeneity of thalamic involvement within the DOC category, regardless of the exact aetiology of the brain injury and extent of lesions in the patients. This suggests that, for all its diversity, DOC samples can inform future clinical studies and pinpoint where in the thalamus brain stimulation therapies may prove more efficacious. Given the heterogeneity both in terms of structural damage and functional dysconnectivity in these types of patient cohorts, we would need to, ideally, begin implementing personalized functional connectome mapping^95^ to achieve improvements in clinical responses from brain stimulation.^95,138,139^ Our differential findings related to the thalamus depending on the type of unconsciousness is especially pertinent, given recent evidence demonstrating distinctive patterns of cortical engagement between arousable unconsciousness (sleep) and unarousable unconsciousness (propofol-induced anaesthesia),^140^ adding to the debate on the shared versus different mechanisms across multiple conditions of unconsciousness.^141^

Our study has several limitations. Firstly, thalamic subdivisions were not segmented at the basis of individual subjects in their native brain space. The processing pipeline we adopted is standard in neuroimaging studies. The atlas we chose should be able to tolerate the normalization error, as it was shown to have high inter-subject consistency and intra-subject reproducibility.^53^ However, we believe it desirable to generate individual-specific parcellations which could provide even higher anatomic accuracy. This may be experimented and validated by future studies. Secondly, we recognize that the healthy volunteers who were given propofol may have been not in in *unconscious state* per se, but rather, an *unresponsive state,* which could be explained by the effect-site concentration and plasma concentration used in our study. Furthermore, given the nature of the patient cohort, segmentation for nuclei may not be perfect in all cases, especially considering the inherent challenges of spatially normalizing DOC patients following brain injury. However, we implemented a rigorous spatial normalization process that brought all images into standard space, facilitated the use of a validated, high-resolution and fine-parcellation thalamic atlas^53^ in a region of interest analysis, and applied a stringent visual quality control; all these steps ensured our segmentation was as accurate as achievable, supporting the robustness of our findings. Finally, we used the datasets from two imaging sites with non-identical scanner hardware and acquisition parameters. We compared the DOC group to the healthy awake participants from the anaesthesia experiment. However, we are confident this does not influence our interpretation of the main results, due to the fact we focused our analysis on between-nuclei comparisons, by which the system variance caused by different scanners should be evened out. Additionally, our results related to the DOC experiment should hold, because they demonstrated a behavioural correspondence which was tested within subsets of the DOC dataset. In fact, previous publications utilizing multi-site datasets have proven the validity of this approach.^28^

Our work which has aimed to identify which thalamic nuclei are more prominent in loss of consciousness could bring diagnostic and therapeutic value for DOC patients. Considering that consciousness depends on the interplay of brain networks,^4,30,41^ we could use localized individualized brain stimulation, in one or multiple nuclei, to induce controlled perturbations in a specific node of the network, which would trigger the propagation of neural activity across proximal and distal brain regions.^142^ In this case, the nuclei which ‘stirred’ the connectivity across the whole brain the most may have the highest chance for neuromodulatory effects following targeted thalamic stimulation. Considering that no clear consensus currently exists on the ideal neural substrates for therapeutic neuromodulation of consciousness,^7^ several functional neuroimaging studies in patients have been able to suggest the involvement of specific brain regions or circuits for targeted stimulation. One study reverted to cortical stimulation in DOC patients and provided the first proof of principle in a sham-controlled randomized double-blind study that non-invasive technique of transcranial direct current stimulation (tDCS) of anterior cortical regions, notably the dorso-lateral prefrontal cortex, can increase CRS-R scores and lead to cognitive gains in MCS patients after brain injury.^143^ Alternatively, central-lateral thalamic stimulation has been demonstrated in a few single-case studies,^24,144^ whereas an alternative to DBS, the non-invasive technique of low-intensity focused ultrasound, targeting the central thalamus has been reported to exhibit clinically significant increases in behavioural responsiveness in two chronic DOC patients.^145,146^ Furthermore, DBS on the central thalamus in a MCS patient exhibited reactivations of dormant functional brain networks; however, increases in consciousness were limited.^147^ Moreover, a single-case study demonstrated the effects of DBS were more long-lasting than those observed with pharmacological interventions, notably zolpidem, albeit the behavioural improvements were still limited.^148^ Until now, however, stimulation studies have been limited, which presses for the need for future work—perhaps, we need to be targeting not one but multiple nuclei in the thalamus for recovery of consciousness, as demonstrated by recent investigations of a novel multiple-target thalamic stimulation paradigm in epilepsy.^149^ Given our findings, one way forward could be to redefine stimulation protocols to add the VLV as a target. Our work, thus, offers the most up-to-date elucidation of consciousness-relevant regions in the thalamus, in the hope of marking an accurate, consistent, and reliable target for stimulation site with DBS for recovery from DOC in the not-so-distant future.

## Conclusions

In summary, this is a systematic investigation of thalamic connectivity involving both healthy anaesthetized and DOC patients, where we establish that (i) anaesthesia and DOC exhibit distinct involvement of thalamic nuclei for loss of consciousness; (ii) specific nuclei, Pu and VLV, account for the greatest functional changes in anaesthesia and DOC, respectively and (iii) distinct cortical networks may contribute to the underlying mechanisms accounting for the functional changes between these thalamic subdivisions and rest of the brain in loss of consciousness. Perhaps most critical of all, we encourage the need for more parcellated explorations into the thalamic mosaic for the purpose of reaching a more unified consensus on the diagnostics and therapies for DOC.

## Supplementary Material

fcag021_Supplementary_Data

## Data Availability

Access to DOC patient data is limited to qualified researchers for non-commercial use to protect patient confidentiality. The UK Health Research Authority assigns responsibility for safeguarding the data to the Chief Investigators of the original studies (Dr. Judith Allanson and Prof. David Menon, or anyone else to whom responsibility is given). To request access, please contact the Data Access Committee: Dr. Judith Allanson (judith.allanson.1@gmail.com), Prof. David Menon (dkm13@cam.ac.uk) or Dr. Emmanuel Stamatakis (eas46@cam.ac.uk). The propofol dataset is publicly accessible via the OpenNeuro data repository (doi: 10.18112/openneuro.ds003171.v2.0.1). The codes produced and used for this work have been uploaded at https://github.com/dorottyaszocs1/Thalamus_in_-un-consciousness_in_pharmacological_and_pathological_states.
