# Supplementary material for: A thalamic perspective of (un)consciousness in pharmacological and pathological states in humans
Source: Brain Commun. 2026 Feb 28;8(2):fcag021. doi: 10.1093/braincomms/fcag021 (PMC13084198; doi:10.1093/braincomms/fcag021)
Supplement: fcag021_Supplementary_Data [file fcag021_supplementary_data.pdf]

Supplementary Material:

Supplementary Fig. 1:

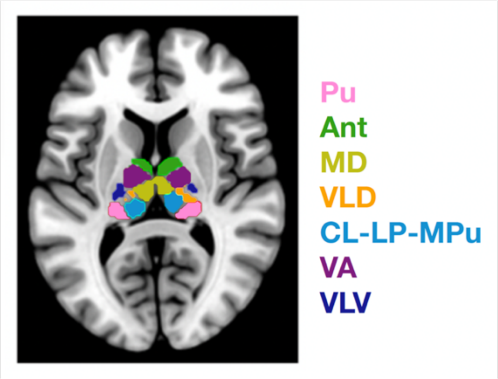

Supplementary Fig. 1: Visualisation of thalamic nuclei. 7 thalamic masks were overlayed for illustration onto standard MNI152 template. Thalamic masks corresponded to the following bilateral nuclei: pulvinar (Pu), anterior (Ant), medio-dorsal (MD), ventral-latero-dorsal (VLD), central-lateral, lateral-posterior, medial-pulvinar group (CL-LP-MPu), ventral-anterior (VA), and ventral-latero-ventral (VLV) (Najdenovska et al., 2018).

Supplementary Fig. 2:

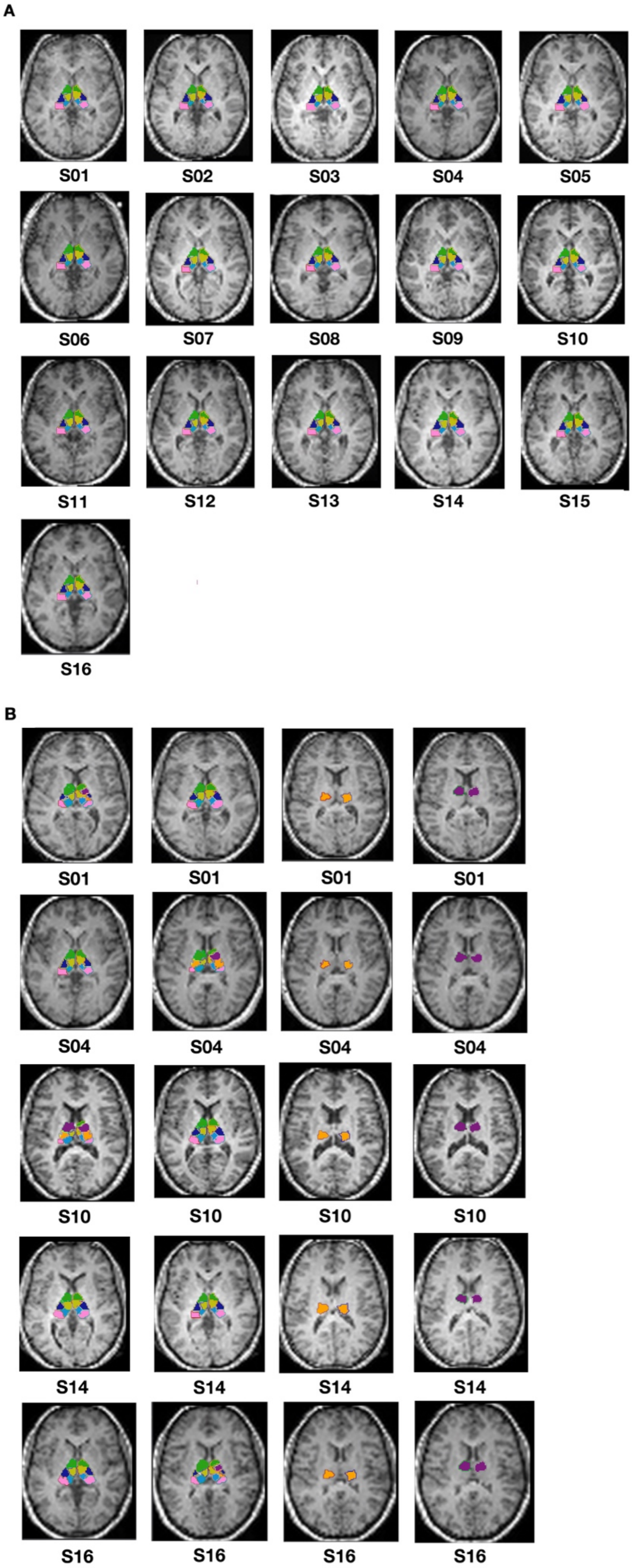

Supplementary Fig 2A and B: Single-subject identification of thalamic subregions in healthy volunteers under anaesthesia (propofol-induced loss of consciousness). The thalamic masks were overlayed for illustration purposes onto the individual's structural scans of all 16 healthy volunteers (S01-S16) (A). As not all individual thalamic nuclei are visible on one slice, additional slices are shown for a subset of volunteers for enhanced visual representation (B).

Supplementary Fig. 3:

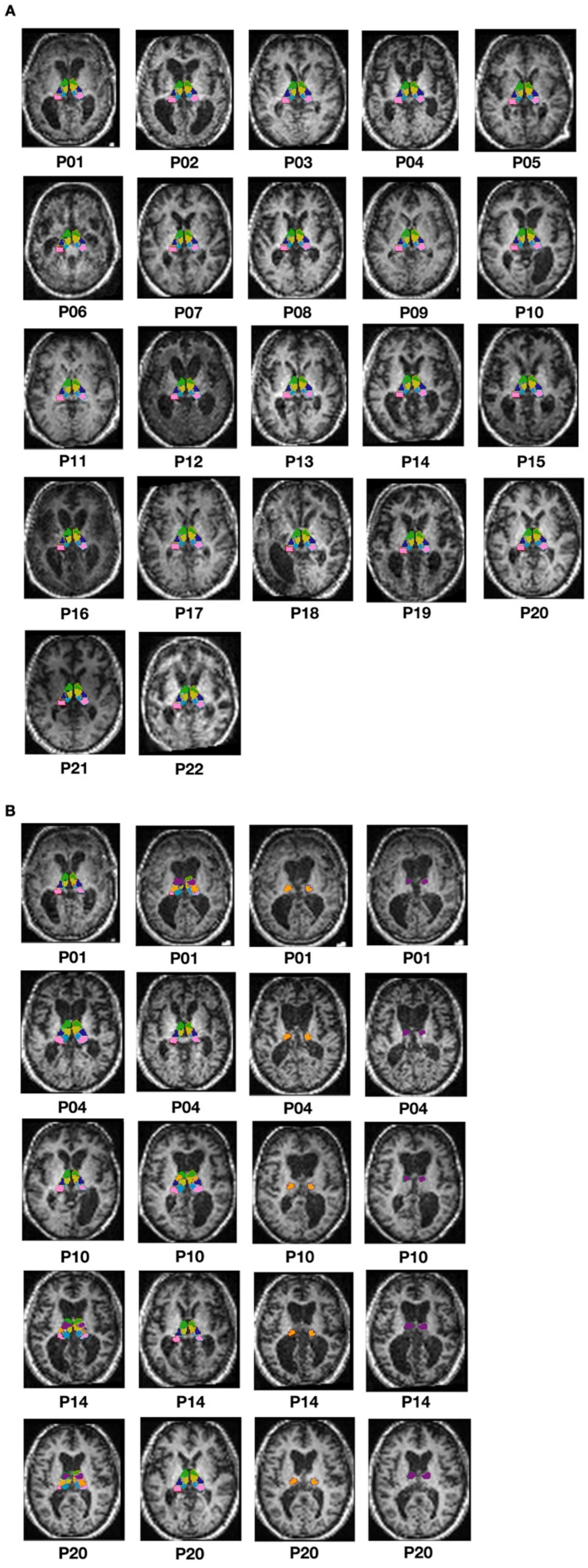

Supplementary Fig 3A and B: Single-subject identification of thalamic subregions in patients with disorders of consciousness (pathological-induced loss of consciousness). The thalamic masks were overlaid for illustration purposes onto the structural scans of all 22 DOC patients (P01-P22) (A). As not all individual thalamic nuclei are visible on one slice, additional slices are shown for a subset of patients for enhanced visual representation (B).

Supplementary Fig. 4:

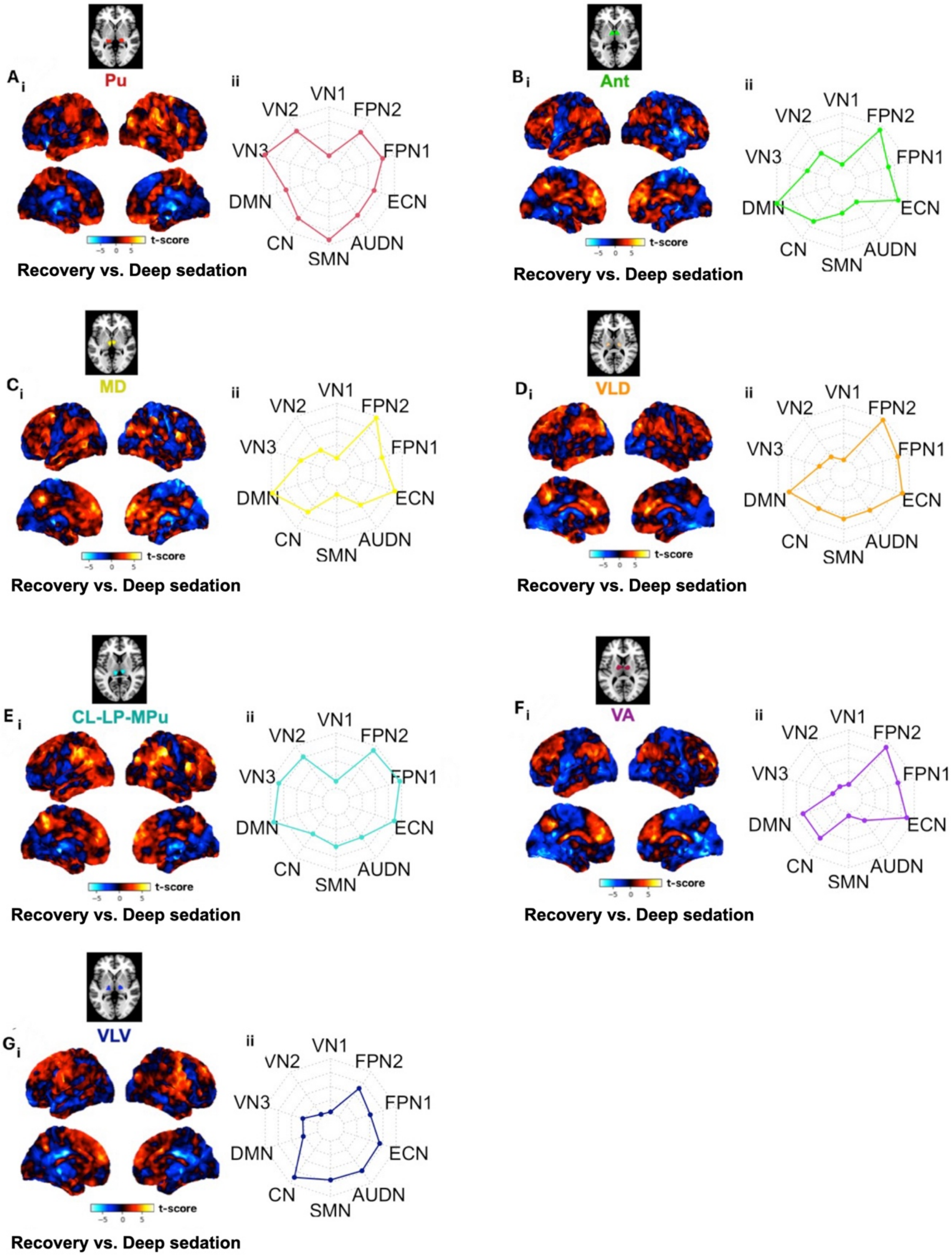

Supplementary Fig. 4: Whole-brain resting-state (rs) functional connectivity (FC) maps in heathy volunteers in recovery of consciousness (from pharmacological i.e. propofol-induced loss of consciousness). Temporal correlations for thalamic seeds were computed for all other voxels in the brain using a general linear model (GLM). For the anaesthesia dataset

where the experimental design is within-subject, FC analyses were first conducted individually, then the individual seed-to-voxel parameter estimate images were entered into group-level analyses. Specifically, paired-sample t tests were used for differences in the participants among the condition of recovery vs. deep sedation. The cortical SPM-t maps from the individuals, both unthresholded and thresholded, were grouped together with a one-sample t-test. The group-level inference of the dataset was corrected for multiple comparisons using random field theory [voxel level threshold of $p<0.005$ (uncorrected) and cluster level $p<0.05$ (FWE-corrected for multiple comparisons)].[78] Results were computed from seed-to-voxel rs-FC analysis across 7 respective thalamic nuclei [bilateral] seeds — Pu, Ant, MD, VLD, CL-LP-MPu, VA, VLV — and the rest of the brain. Colour bar denotes the strength of the t-statistic. Unthresholded t-maps of the recovery vs. deep sedation contrast are shown (**Ai, Bi, Ci, Di, Ei, Fi, Gi**). Radar plots indicate the intrinsic connectivity network (ICN) spatial involvement (ICNi) of brain regions in the recovery vs. deep sedation contrast i.e. shows the voxel overlap between the FC results and canonical ICNs (**Aii, Bii, Cii, Dii, Eii, Fii, Gii**). These canonical ICNs were defined by an atlas[79] containing 10 well-matched resting-state networks (RSN) from the *ICN_atlas* toolbox (https://www.nitrc.org/projects/icn_atlas/).[80] Description of ICN-RSN atlas: VN 1, 2, and 3 ("*visual network*"): medial, occipital pole, and lateral visual areas; DMN ("*default mode network*"): medial parietal (precuneus and posterior cingulate), bilateral inferior–lateral–parietal, and ventromedial frontal cortex; CN ("*cerebellum network*"): cerebellum; SMN ("*sensorimotor network*"): supplementary motor area, sensorimotor cortex, and secondary somatosensory cortex; AUDN ("*auditory network*"): superior temporal gyrus, Heschl's gyrus, and posterior insular. It includes primary and association auditory cortices; ECN ("*executive control network*"): medial–frontal areas, including anterior cingulate and paracingulate; FPN1 and 2 ("*frontoparietal network*"): frontoparietal areas; these are the only maps to be strongly lateralised. In addition, FPN1 corresponds strongly to perception–somesthesis–pain, and FPN2 to cognition–language paradigms, consistent with Broca's and Wernicke's areas.[58] Furthermore, to classify patterns of thalamo-cortical rs-FC findings based on network involvement (ICNi) with respective to individual nuclei, we performed a hierarchical-based clustering algorithm in RStudio (https://www.r-project.org/).

Supplementary References:

Najdenovska E, Alemán-Gómez Y, Battistella G, et al. In-vivo probabilistic atlas of human thalamic nuclei based on diffusion- weighted magnetic resonance imaging. *Sci Data*. 2018;5:180270. doi:10.1038/sdata.2018.270
